# Three new LmbU targets outside *lmb* cluster inhibit lincomycin biosynthesis in *Streptomyces lincolnensis*

**DOI:** 10.1186/s12934-023-02284-y

**Published:** 2024-01-03

**Authors:** Yue Mao, Xianyan Zhang, Tianyu Zhou, Bingbing Hou, Jiang Ye, Haizhen Wu, Ruida Wang, Huizhan Zhang

**Affiliations:** 1 State Key Laboratory of Bioreactor Engineering, East China University of Science and Technology, Shanghai, China; 2 Department of Applied Biology, School of Biotechnology, East China University of Science and Technology, Shanghai, China

**Keywords:** *Streptomyces lincolnensis*, Lincomycin biosynthesis, Transcriptional regulation, LmbU, Cluster-situated regulatory gene(s)

## Abstract

**Background:**

Antibiotics biosynthesis is usually regulated by the cluster-situated regulatory gene(s) (CSRG(s)), which directly regulate the genes within the corresponding biosynthetic gene cluster (BGC). Previously, we have demonstrated that LmbU functions as a cluster-situated regulator (CSR) of lincomycin. And it has been found that LmbU regulates twenty non-*lmb* genes through comparative transcriptomic analysis. However, the regulatory mode of CSRs’ targets outside the BGC remains unknown.

**Results:**

We screened the targets of LmbU in the whole genome of *Streptomyces lincolnensis* and found fourteen candidate targets, among which, eight targets can bind to LmbU by electrophoretic mobility shift assays (EMSA). Reporter assays in vivo revealed that LmbU repressed the transcription of *SLINC_0469* and *SLINC_1037* while activating the transcription of *SLINC_8097*. In addition, disruptions of *SLINC_0469*, *SLINC_1037*, and *SLINC_8097* promoted the production of lincomycin, and qRT-PCR showed that SLINC_0469, SLINC_1037, and SLINC_8097 inhibited transcription of the *lmb* genes, indicating that all the three regulators can negatively regulate lincomycin biosynthesis.

**Conclusions:**

LmbU can directly regulate genes outside the *lmb* cluster, and these genes can affect both lincomycin biosynthesis and the transcription of *lmb* genes. Our results first erected the cascade regulatory circuit of LmbU and regulators outside *lmb* cluster, which provides the theoretical basis for the functional research of LmbU family proteins.

**Supplementary Information:**

The online version contains supplementary material available at 10.1186/s12934-023-02284-y.

## Introduction

Streptomycetes are high G+C, filamentous Gram-positive bacteria. In order to cope with the complex and changeable living environment, streptomycetes evolved a set of protective mechanisms with competitive advantages [1], producing a large number of secondary metabolites, such as antibiotics with high medical value [2, 3]. Antibiotic biosynthesis is stringently controlled by precise and pyramidal regulatory cascades [4]. *Streptomyces* can monitor the environmental conditions, growing states, population density, and so on, and then secrete and sense specific signal small molecules named autoregulators, including γ-butyrolactones (GBLs), antibiotics and biosynthetic intermediates [5–7]. Then, the receptors of autoregulators respond and transmit these signal inputs to corresponding transcriptional regulators of secondary metabolite, thereby regulating antibiotics biosynthesis. Transcriptional regulators in *Streptomyces* are usually classified as global/pleiotropic regulators and CSRs [8]. The global/pleiotropic regulators can not only regulate the biosynthesis of secondary metabolites, but also affect the morphological differentiation of *Streptomyces* [9, 10]. The BGC of each antibiotic usually includes one or more CSRs, which are at the bottom of the secondary metabolic regulatory network, and directly regulate transcription of the corresponding antibiotics biosynthetic genes, thereby regulating antibiotics biosynthesis [11, 12].

Many studies have shown that the targets of CSRs are not limited to the gene cluster in which they are situated, they may also located in disparate antibiotic BGCs, forming cross-regulation [13]. For instance, FscRI regulates candicidin biosynthesis as well as antimycin biosynthesis in *Streptomyces albus* S4 [14]. GdmRIII up-regulates the production of geldanamycin and down-regulates that of elaiophylin by affecting transcription of the genes in both gene clusters in *Streptomyces autolyticus* CGMCC0516 [15]. JadR1 can not only activate jadomycin biosynthesis by directly binding to the promoter region of *jadJ*, but also repress chloramphenicol biosynthesis by directly binding to the promoter region of *cmlJ* in chloramphenicol BGC in *Streptomyces venezuelae* [16, 17]. Similar examples are also found in coordinated cephamycin C and clavulanic acid biosynthesis regulated by CcaR in *Streptomyces clavuligerus* [18], RED, ACT and CDA biosynthesis regulated by RedZ in *Streptomyces coelicolor* [19], avermectin and oligomycin biosynthesis regulated by AveR in *Streptomyces avermitilis* [20], and rapamycin and elaiophylin biosynthesis regulated by RapH in *Streptomyces rapamycinicus* [11]. Though cross-regulation of disparate antibiotics by one CSR has been reported many times, screening of the targets of CSRs outside the BGSs is barely reported. Li et al. [21] showed that NemR functions as a pleiotropic regulator, which not only activates transcription of the genes within nemadectin BGC, but also regulates four targets outside the BGC in *Streptomyces cyaneogriseus* ssp. *noncyanogenus*.

Lincomycin, one of the lincosamide antibiotics, was isolated from a soil-derived Gram-positive bacterium *Streptomyces lincolnensis* in 1962 [22]. The 32-kb BGC of lincomycin (*lmb*) contains twenty-five structural genes [23, 24], two resistance genes [25], one dual-functional gene [26] and one CSR [27]. Along with the complete elucidation on the biosynthesis route of lincomycin [23, 24], several studies have been reported to explore the regulation mechanism of lincomycin biosynthesis. For the first time, we identified a novel CSR LmbU, which positively regulates lincomycin biosynthesis [27, 42]. Subsequently, several other regulators that are located outside the *lmb* cluster and modulate lincomycin biosynthesis, including AdpA, BldA, BldD, GlnR, RamR, AflQ1-Q2, Rex, SLCG_2919, SLCG_Lrp, σ^L^ and AtrA etc., have been characterized [28–40].

In our previous study, we characterized LmbU as a CSR of lincomycin, and demonstrated that LmbU homologues are widely found in actinomycetes, indicating LmbU might regulate other target genes except *lmb* genes. Here, we demonstrated that LmbU functions as a pleiotropic regulator, which negatively regulates transcription of *SLINC_0469* and *SLINC_1037*, while positively regulates transcription of *SLINC_8097*. In addition, we showed that *SLINC_0469*, *SLINC_1037* and *SLINC_8097* can all inhibit the production of lincomycin by repressing transcription of *lmb* genes.

## Material and methods

### Bacterial strains, plasmids and culture conditions

Bacterial strains and plasmids used in this study are listed in Table 1. *Escherichia coli* strains and *S. lincolnensis* strains were described in our previous study [27]. Moreover, YEME medium (10 *g*/L yeast extract, 5 *g*/L polypeptone, 10 *g*/L glucose, 3 *g*/L malt extract, 5 mM MgCl_2_•2H_2_O, 340 *g*/L sucrose) was used for preparation of *S. lincolnensis* mycelium for conjugation, ISP4 medium (10 *g*/L soluble starch, 1 *g*/L K_2_HPO_4_, 5 *g*/L MgSO_4_•7H_2_O, 1 *g*/L NaCl, 2 *g*/L (NH_4_)_2_SO_4_, 2 *g*/L CaCO_3_, 15 *g*/L Agar, 0.001 *g*/L FeSO_4_•7H_2_O, 0.001 *g*/L MnCl_2_•4H_2_O, 0.001 *g*/L ZnSO_4_•7H_2_O, 0.02 M MgCl_2_) was used for conjugation of *E. coli* and *S. lincolnensis*.

**Table 1:** Strains and plasmids

Strains or plasmids	Description	Source or reference
Strains		
*E. coli*		
JM83	F’, ara, Δ(*lac-pro* AB), *rpsL*, (Str^r^), Φ80, *lacZ*ΔM15	[27]
ET12567/pUZ8002	*dam-13*::Tn9 *dcm-6 hsdM*; containing the non-transmissible RP4 derivative plasmid pUZ8002	[27]
S17-1	*recA*, *pro*, *hsdR*, RP4-2-Tc::Mu-Km::Tn7	[37]
*S. lincolnensis*		
NRRL 2936	Wild-type (WT), lincomycin producer	NRRL, USA
Δ*lmbU*	NRRL 2936 with in-frame deletion of *lmbU*	This study
WT/p0469TE	NRRL 2936 attBφC31::p0469TE	This study
Δ*lmbU*/p0469TE	Δ*lmbU* attBφC31::p0469TE	This study
WT/p1037TE	NRRL 2936 attBφC31::p1037TE	This study
Δ*lmbU*/p1037TE	Δ*lmbU* attBφC31::p1037TE	This study
WT/p8097TE	NRRL 2936 attBφC31::p8097TE	This study
Δ*lmbU*/p8097TE	Δ*lmbU* attBφC31::p8097TE	This study
Δ*SLINC_0469*	NRRL 2936 with in-frame deletion of *SLINC_0469*	This study
Δ*SLINC_1037*	NRRL 2936 with in-frame deletion of *SLINC_1037*	This study
Δ*SLINC_8097*	NRRL 2936 with in-frame deletion of *SLINC_8097*	This study
C*SLINC_0469*	*SLINC_0469* complementation strain	This study
C*SLINC_1037*	*SLINC_1037* complementation strain	This study
C*SLINC_8097*	*SLINC_8097* complementation strain	This study
Plasmids		
pLU-1	*lmbU* cloned in *Nde*I/*Eco*RI sites of pET-28a ( +), for LmbU expression	[27]
pKCcas9dO	*actII-orf4*-specific guide-RNA, homologous arms flanking *actII-orf4*, *aac(3)IV*, pSG5	[41]
pKCcas9dlmbU	*lmbU*-specific guide-RNA, homologous arms flanking *lmbU*, *aac(3)IV*, pSG5	This study
pIB139	Integrative vector based on φC31 integrase, ermE*p	[27]
pPU139	pIB139 carrying *lmbU* gene under its own promoter	This study
pATE152	pSET152 derivative carrying *xylTE* gene under *lmbAp* promoter	[42]
pSET152	Integrative vector based on φC31 integrase	[43]
p0469TE	pSET152 carrying *xylTE* gene under the control of *SLINC_0469p*	This study
p1037TE	pSET152 carrying *xylTE* gene under the control of *SLINC_1037p*	This study
p8097TE	pSET152 carrying *xylTE* gene under the control of *SLINC_8097p*	This study
pKCcas9d0469	*SLINC_0469*-specific guide-RNA, homologous arms flanking *SLINC_0469*, *aac(3)IV*, pSG5	This study
pKCcas9d1037	*SLINC_1037*-specific guide-RNA, homologous arms flanking *SLINC_1037*, *aac(3)IV*, pSG5	This study
pKCcas9d8097	*SLINC_8097*-specific guide-RNA, homologous arms flanking *SLINC_8097*, *aac(3)IV*, pSG5	This study

### Expression and purification of His_6_-LmbU

The expression plasmid pLU-1 [27] was transformed into *E. coli* BL21 (DE3), and used for His_6_-LmbU expression. The strain was cultivated in 100 mL LB medium at 37 ℃ until OD_600_ reached about 0.6, then 1 mM Isopropyl β-D-1-thiogalactopyranoside was added. After overnight cultivation at 16 ℃, the cells were washed twice and suspended in PBS buffer (0.1 M phosphate buffer solution, pH 7.5). Total proteins were released by sonication and His_6_-LmbU was purified using nickeliminodiacetic acid–agarose chromatography (Weishibohui, Beijing, China). After dialysis and concentration, the purified protein was stored in binding buffer (10 mM Tris–HCl [pH 8.0], 1 mM EDTA, 0.2 mM dithiothreitol, 20 μg/mL bovine serum albumin, 1.2% glycerol).

### Electrophoretic mobility shift assay (EMSA)

DNA probes of around 200 bp containing the binding sites of LmbU were amplified via two rounds of PCR. Firstly, primer pairs UBS-X-F/R (X indicates the numbers of the 14 putative targets of LmbU) were used to amplify the cold probes without biotin. Then, biotin-labeled primer EMSA-B* was used for the second-round PCR to generate the labeled probes. The probe prepared by primer pair nag-F/R was used as a negative control. EMSAs were performed as described previously [27] using chemiluminescent EMSA kits (Beyotime, Shanghai, China) with some modification in binding buffer, which included 10 mM Tris–HCl (pH 8.0), 1 mM EDTA, 0.2 mM dithiothreitol, 20 *g*/mL bovine serum albumin, 1.2% glycerol, and 50 μg/mL poly(dI-dC) [27]. EMSAs performed with 200-fold excesses of specific or nonspecific cold probes were added as controls to confirm the specificity of the band shifts.

All primer pairs used in this study are listed in Additional file 1: Table S1.

### Construction of *lmbU* disruption strain Δ*lmbU*

To construct a *lmbU* disruption strain, the internal region of *lmbU* (465 bp) was deleted via a CRISPR/Cas9-based genetic editing method [41]. The *lmbU*-specific single-molecule-guide RNA (sgRNA) was amplified by PCR using the primer pair sgUF/R with pKCcas9dO as template. Upstream (1.2 kb) and downstream (1.2 kb) homologous arms of *lmbU* were amplified by PCR using primer pairs uU-F/R and dU-F/R, respectively. The *lmbU*-specific deletion cassette was assembled with the above three DNA fragments by using overlapping PCR. Subsequently, the deletion cassette was digested with *Spe*I and *Hin*dIII (Thermo Fisher, Waltham, MA, USA), and ligated into the corresponding sites of pKCcas9dO. The resulting plasmid pKCcas9dlmbU was introduced into *S. lincolnensis* NRRL 2936 by conjugation, using *E. coli* S17-1 as a donor. The conjugants were selected with nalidixic acid and apramycin, and then identified by PCR using the primer pair JDU-F/R and DNA sequencing. The pKCcas9dlmbU plasmid was eliminated through a few rounds of streak cultivation in YEME medium at 37 ℃, which was identified by PCR using the primer pair CR1/2.

### Catechol dioxygenase activity analysis

The regions upstream (relative to the translational start site) of *SLINC_0469* (−578 to −1), *SLINC_1037* (−471 to −1) and *SLINC_8097* (−427 to −1) were amplified using primer pairs p0469-F/R, p1037-F/R and p8097-F/R respectively. The reporter genes *xylTE* were amplified by PCR using primer pair pAxyl-3/4, with pATE152 as a template. Two DNA fragments (promoter region and reporter gene) were cloned into the *Pvu*II (Thermo Fisher) site of the integrative plasmid pSET152 using Super Efficiency Fast Seamless Cloning kits (DoGene, Shanghai, China), resulting in reporter plasmids p0469TE, p1037TE and p8097TE. Then, the reporter plasmids were transferred into the wild-type strain NRRL 2936 and the *lmbU* disruption strain Δ*lmbU*, to construct the reporter strains WT/p0469TE, WT/p1037TE, WT/p8097TE, Δ*lmbU*/p0469TE, Δ*lmbU*/p1037TE and Δ*lmbU*/p8097TE.

Catechol dioxygenase activity analysis was performed as described previously [28]. Briefly, the reporter strains were cultivated in YEME medium at 28 ℃ for 1 day, then the cells were harvested and lysed by sonication. An appropriate amount of cell extract was added to the assay buffer (100 mM potassium phosphate [pH 7.5], 1 mM catechol), and the optical density at 375 nm was detected per minute. The rate of change per minute per milligram of absorbance was calculated as catechol dioxygenase activity.

### Bioinformatics analysis (Functional domain analysis, sequence alignment and structure modeling)

Functional domain analysis was performed by BlastP in National Center for Biotechnology Information (NCBI) (https://blast.ncbi.nlm.nih.gov/Blast.cgi?PROGRAM=blastp&PAGE_TYPE=BlastSearch&LINK_LOC=blasthome). Sequence alignment was analyzed using the online software ESPript 3.0 (https://espript.ibcp.fr/ESPript/cgi-bin/ESPript.cgi). Structure modeling was constructed using the online software SWISS MODEL (https://swissmodel.expasy.org/interactive).

### Construction of *SLINC_0469*, *SLINC_1037* and *SLINC_8097* disruption, complementation and overexpression strains.

To construct the *SLINC_0469* disruption strain (Δ*SLINC_0469*), the same CRISPR/Cas9-based genetic editing method was carried out as construction of Δ*lmbU* with some modification in construction of disruption plasmids. For instance, to construct Δ*SLINC_0469*, the upstream and downstream homologous arms of *SLINC_0469* were amplified by PCR using primer pairs u-0469-F/R and d-0469-F/R, respectively. Specific sgRNA was added to upstream homologous arm by PCR using the primer pair sg-0469/u-0469-R. The above two DNA fragments (sgRNA-containing upstream and downstream homologous arms) were cloned into the *Spe*I/*Hin*dIII (Thermo Fisher) sites of pKCcas9dO using Super Efficiency Fast Seamless Cloning kits (DoGene), resulting in disruption plasmid pKCcas9d0469. The primer pair JD-0469-F/R was used to identify the conjugants selected with nalidixic acid and apramycin, and the primer pair CR1/2 was used to verify the elimination of the disruption plasmid.

To construct *SLINC_0469* complementation and overexpression strains, the *SLINC_0469* expression cassette containing *SLINC_0469*_*p*_ promoter and SLINC_0469 encoding gene was amplified by PCR using the primer pair p0469-F/0469-R. The obtained cassette was cloned into the *Pvu*II (Thermo Fisher) sites of pIB139 by using Super Efficiency Fast Seamless Cloning kits (DoGene). The resulting plasmid pIBpN0469 was introduced into Δ*SLINC_0469* by conjugation, resulting in *SLINC_0469* complementation strain C*SLINC_0469* and ocerexpression strain O*SLINC_0469*.

Constructions of *SLINC_1037* and *SLINC_8097* disruption, complementation and overexpression strains were carried out as the similar procedures of *SLINC_0469*.

### Lincomycin bioassay analysis

Lincomycin bioassay analysis was carried out as described in our previous work [27]. FM2 medium (20 *g*/L lactose, 20 *g*/L glucose, 10 *g*/L corn steep liquor, 10 *g*/L polypeptone, 4 *g*/L CaCO_3_, pH 7.0) was used for fermentation cultivation. *Micrococcus luteus* 28001 was used as an indicator strain, and the concentrations of samples were measured according to the lincomycin standard curves.

Three biological independent experiments were done for the analytical procedures. Error bars indicated means ± standard deviations.

### RNA extraction and quantitative real-time PCR (qRT-PCR)

The strains were cultured in FM2 medium for 2 days, and then RNA was extracted by the method using TRIzol (Thermo Fisher) [30]. The trace amount of DNA was removed through incubation with RNase-free DNase I (TaKaRa, Dalian, China) at 28 ℃, and the obtained RNA was analyzed using NanoDrop 2000 (Thermo Fisher). 1 μg RNA was used to synthesize the cDNA using reverse transcription M-MLV (RNase-free) kits (TaKaRa). qRT-PCR was performed with SYBR green PCR master mix (ToYoBo, Shanghai, China) as described previously [27]. PCR was carried out in triplicate for each sample. The transcriptional level of *hrdB* was used as a positive internal control to normalize the transcriptional levels of target genes, which were measured by the threshold cycle (2^−ΔΔ*CT*^) method [44].

## Results

### Potential targets of LmbU were found in the genome of *S. lincolnensis*

We have previously demonstrated that the conserved palindrome sequence 5ʹ-CGCCGGCG-3ʹ allows LmbU to bind to the promoter regions of *lmbA* and *lmbW* [27]. To explore the potential regulatory targets of LmbU and reduce non-specific bindings, we conducted a genome-wide scan of *S. lincolnensis* using the extended motif (5ʹ-TCGCCGGCGA-3ʹ) derived from the binding motif of the *lmbA* promoter region. A total of 176 conserved sequences were found throughout the genome, among which 54 were located in the potential regulatory regions (– 600 to + 100 bp relative to the putative translational start site, and not located inside the operon). Subsequently, 14 candidate targets which may be relevant to lincomycin biosynthesis were selected, including 4 regulators, 5 transporters or resistance-related proteins, 2 σ factors, and 3 other functional proteins (Table 2).

**Table 2:** Candidate target genes of LmbU

Genes	Products	Putative function	Position of putative LmbU site to TRSS^a^
***SLINC_0469***	**WP_067426176.1, 988 aa**	**LAL family transcriptional regulator**	**−316 – −307**
***SLINC_1037***	**WP_079164420.1, 635 aa**	**AcoR family transcriptional regulator**	**−281 – −272**
*SLINC_4499*	WP_067445653.1, 186 aa	TetR family transcriptional regulator	+89 – +98
***SLINC_8097***	**WP_067443797.1, 301 aa**	**AraC family transcriptional regulator**	**−365 – −356**
***SLINC_0585***	**WP_067426389.1, 510 aa**	**MFS transporter**	**−172 – −163**
*SLINC_1746*	WP_067429133.1, 275 aa	ABC transporter substrate-binding protein	−176 – −167
***SLINC_6382***	**WP_067441376.1, 327 aa**	**ABC transporter permease**	**−428 – −419**
*SLINC_RS37445*	WP_067446118.1, 135 aa	vicinal oxygen chelate family protein	−322 – −313
***SLINC_7298***	**WP_067443078.1, 478 aa**	**MFS transporter**	**−346 – −337**
*SLINC_6232*	WP_067445952.1, 511 aa	RNA polymerase sigma factor	−549 – −540
***SLINC_6570***	**WP_067441886.1, 201 aa**	**RNA polymerase sigma factor**	**−549 – −540**
***SLINC_1077***	**WP_067427571.1, 256 aa**	**methyltransferase**	**−492 – −483**
*SLINC_4366*	WP_067436406.1, 574 aa	methionine–tRNA ligase	−215 – −206
*SLINC_6271*	WP_067441084.1, 194 aa	PaaI family thioesterase	−281 – −272

^**a**^TRSS translational start site

The positive target genes confirmed by EMSA were indicated by bold type

### LmbU binds to the promoter regions of 8 target genes directly

**Fig. 1:**
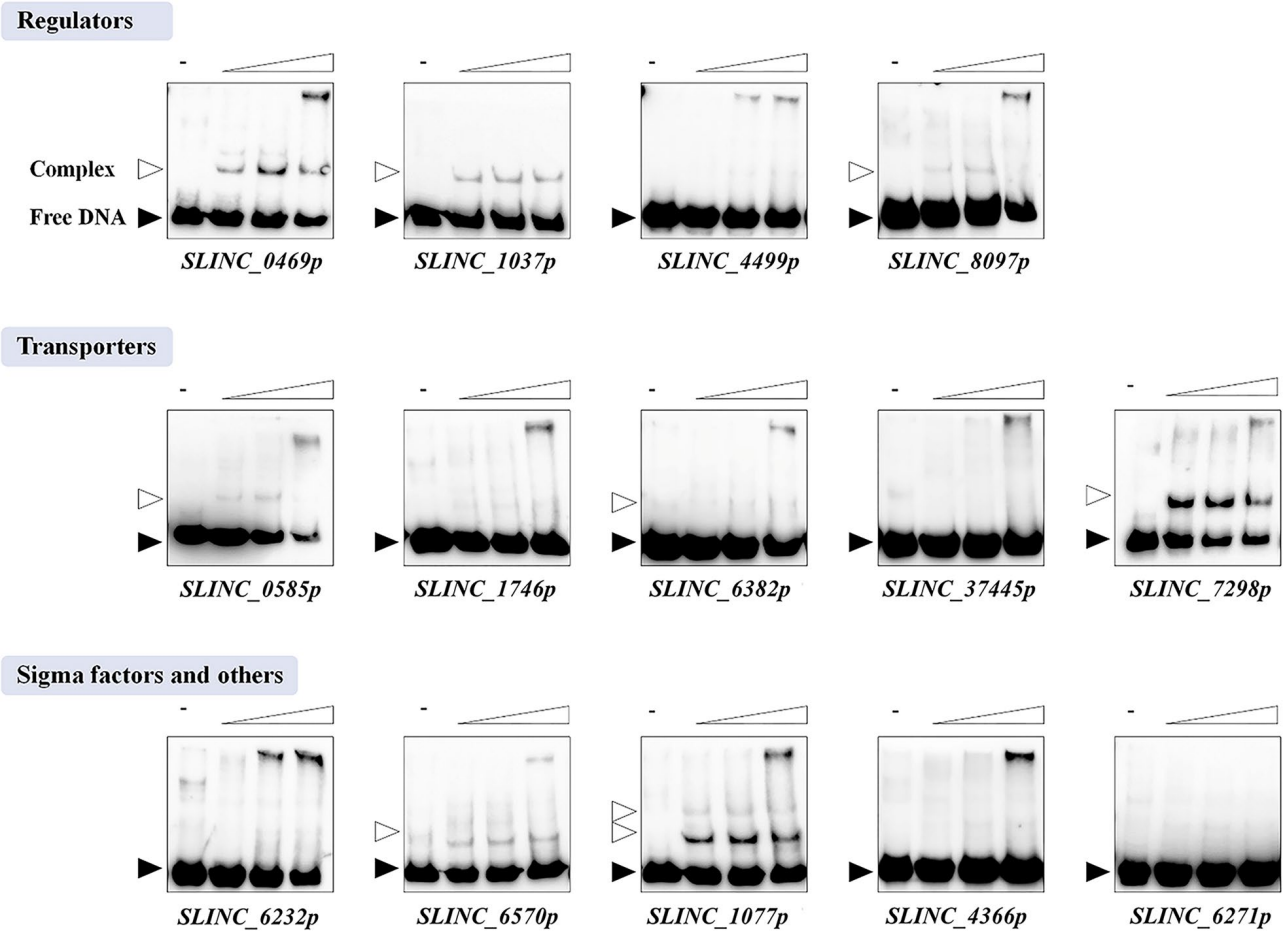
Identification of the binding activities of LmbU to the putative targets. Biotin-labeled probes (5 ng) were incubated with His_6_-LmbU of increasing concentrations (0, 3.2, 6.4 and 9.6 µM). The free probes and DNA–protein complexes are indicated by filled triangles and hollow triangles respectively

In order to investigate whether LmbU can bind to the above 14 targets, EMSAs were carried out with purified His_6_-LmbU and the DNA probes of candidate targets. The results showed that His_6_-LmbU could obviously bind to the promoter regions of 8 genes in a concentration-dependent manner (Fig. 1). The deduced products of the 8 target genes were as follows: LAL family transcriptional regulator (encoded by *SLINC_0469*), AcoR family transcriptional regulator (encoded by *SLINC_1037*), AraC family transcriptional regulator (encoded by *SLINC_8097*), MFS transporter (encoded by *SLINC_0585*), ABC transporter permease (encoded by *SLINC_6382*), MFS transporter (encoded by *SLINC_7298*), RNA polymerase σ factor (encoded by *SLINC_6570*) and methyltransferase (encoded by *SLINC_1077*).

**Fig. 2:**
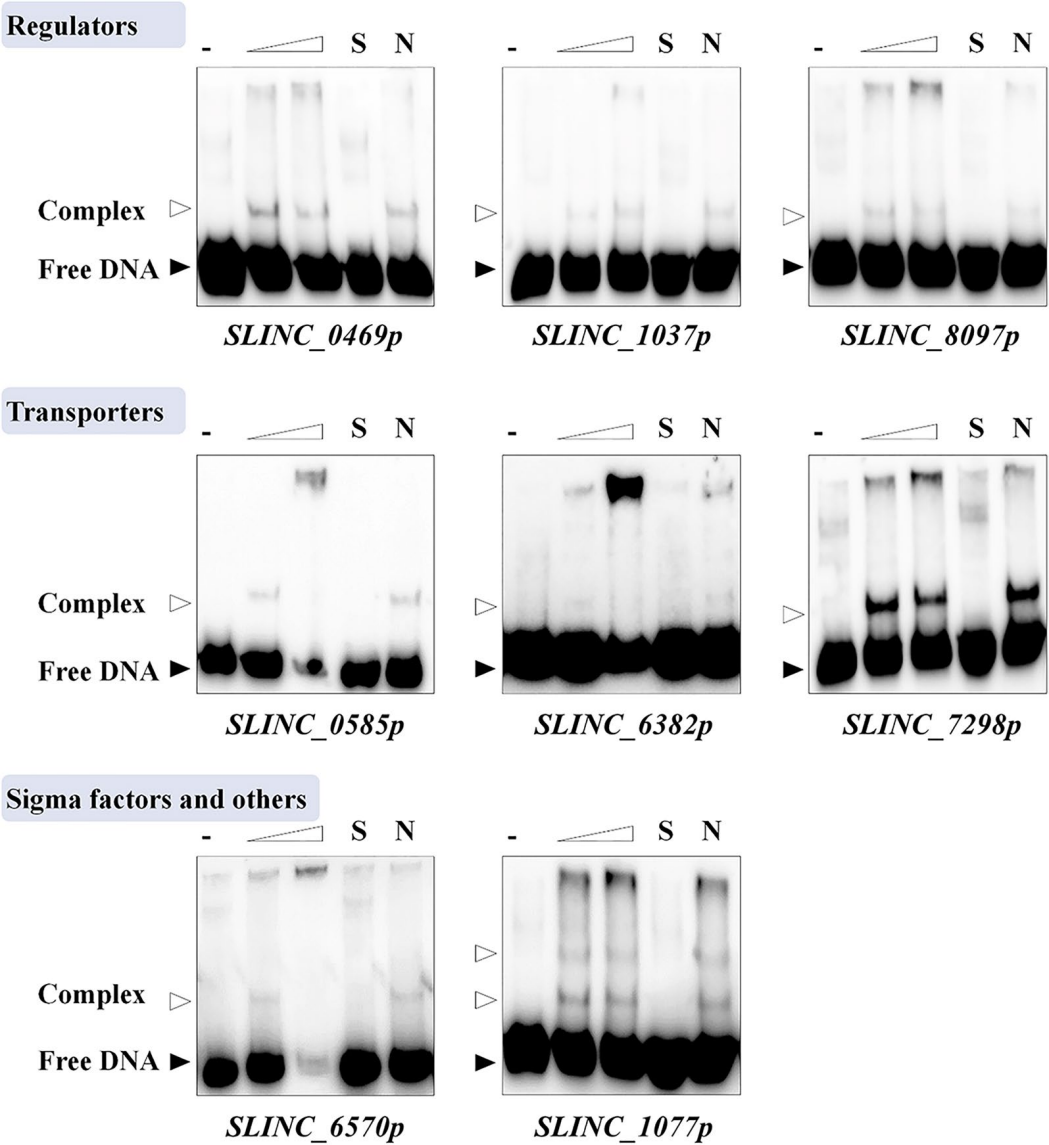
EMSAs of LmbU with promoter regions of target genes. Biotin-labeled probes (5 ng) were incubated with His_6_-LmbU of increasing concentrations (0, 6.4 and 9.6 µM). The free probes and DNA–protein complexes are indicated by filled triangles and hollow triangles respectively. 200-fold excess of specific (S) or nonspecific (N) unlabeled probes were used as competitors of the labeled probes

Subsequently, competition experiments were introduced into EMSAs to confirm the binding specificity of His_6_-LmbU to the above 8 targets. In the presence of 6.4 μM His_6_-LmbU, the retardant bands of all 8 targets were significantly weakened when 200-fold excesses of unlabeled specific DNA were added, but did not change when 200-fold excesses of unlabeled nonspecific DNA (a negative probe that cannot bind to His_6_-LmbU, Additional file 1: Fig. S1) were added. These data demonstrated that LmbU can directly and specifically bind to the promoter regions of the above 8 target genes (Fig. 2), including 3 regulators, 3 transporters, 1 σ factor and 1 other functional protein. However, the binding affinities of LmbU with different probes are diverse. LmbU has the highest binding affinity with the probe *SLINC_7298p*, while the weakest binding affinity with the probe *SLINC_6382p*. Besides, two retardant bands were observed when His_6_-LmbU bound to *SLINC_1077p*, indicating the regulatory models of LmbU to these targets may be different.

### LmbU represses the promoters of *SLINC_0469* and *SLINC_1037* and activates the promoter of* SLINC_8097 *in vivo

As we know, antibiotics biosynthesis is strictly controlled by accurate and sophisticated regulatory networks. Through the above studies, we revealed that three regulatory genes may be regulated by LmbU. To investigate the regulation of LmbU to the three regulator genes in vivo, we firstly constructed a *lmbU* disruption strain Δ*lmbU* by using a CRISPR/Cas9-based genetic editing method (Additional file 1: Fig. S2). Then, the WT and Δ*lmbU* strains were chosen for qRT-PCR assays to analyze the effects of LmbU on the transcription of the three target genes. However, transcriptional levels of *SLINC_1037* and *SLINC_8097* were not enough for quantitative analysis (data not shown).

**Fig. 3:**
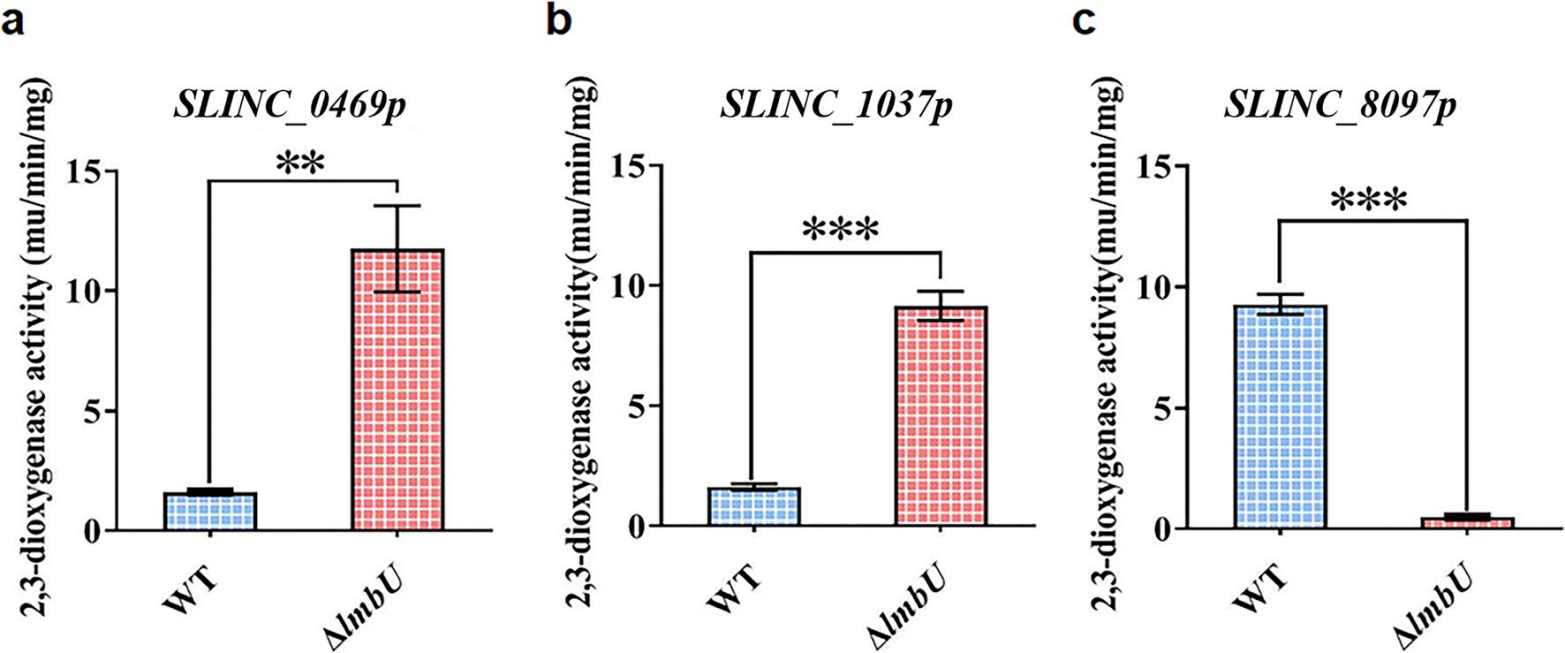
Catechol dioxygenase activity assays of strains WT and Δ*lmbU* with corresponding reporter plasmids. 3a-3c represent enzyme activities of XylE controlled by *SLINC_0469p*, *SLINC_1037p*, and *SLINC_8097p* respectively. The results were achieved from three independent experiments. **P < 0.01; ***P < 0.001

Therefore, we performed *xylTE* reporter assays, using the catechol dioxygenase and cofactor genes (*xylTE*) as a reporter. As results, enzyme activities of XylE controlled by *SLINC_0469p* and *SLINC_1037p* exhibited sevenfold and sixfold increase in Δ*lmbU* compared to that in WT, respectively (Fig. 3a and b), suggesting that LmbU represses the promoters of *SLINC_0469* and *SLINC_1037 *in vivo. In contrast, enzyme activities of XylE controlled by *SLINC_8097p* showed 19-fold decrease in Δ*lmbU* compared to that in WT (Fig. 3c), suggesting that LmbU activates the promoter of *SLINC_8097 *in vivo.

### SLINC_0469 negatively regulates lincomycin biosynthesis

The gene *SLINC_0469* is 2,967 bp, and encodes a protein containing 988 amino acids, which belongs to a large ATP-binding regulator of the LuxR family (LAL) transcriptional regulator. Sequence alignment showed that N-terminal of SLINC_0469 has an AAA + (ATPases Associated with a wide variety of Activities) domain with ATPase activity, which contains conserved Walker A motif (A/G-X4-G-K-S/T, X indicates any amino acids) and Walker B motif (hhhhhDD, h indicates hydrophobic amino acids) (Additional file 1: Fig. S3a, b). C-terminal of SLINC_0469 has a DNA-binding domain (DBD) of the helix-turn-helix (HTH) structure of the LuxR family (Additional file 1: Fig. S3c).

**Fig. 4:**
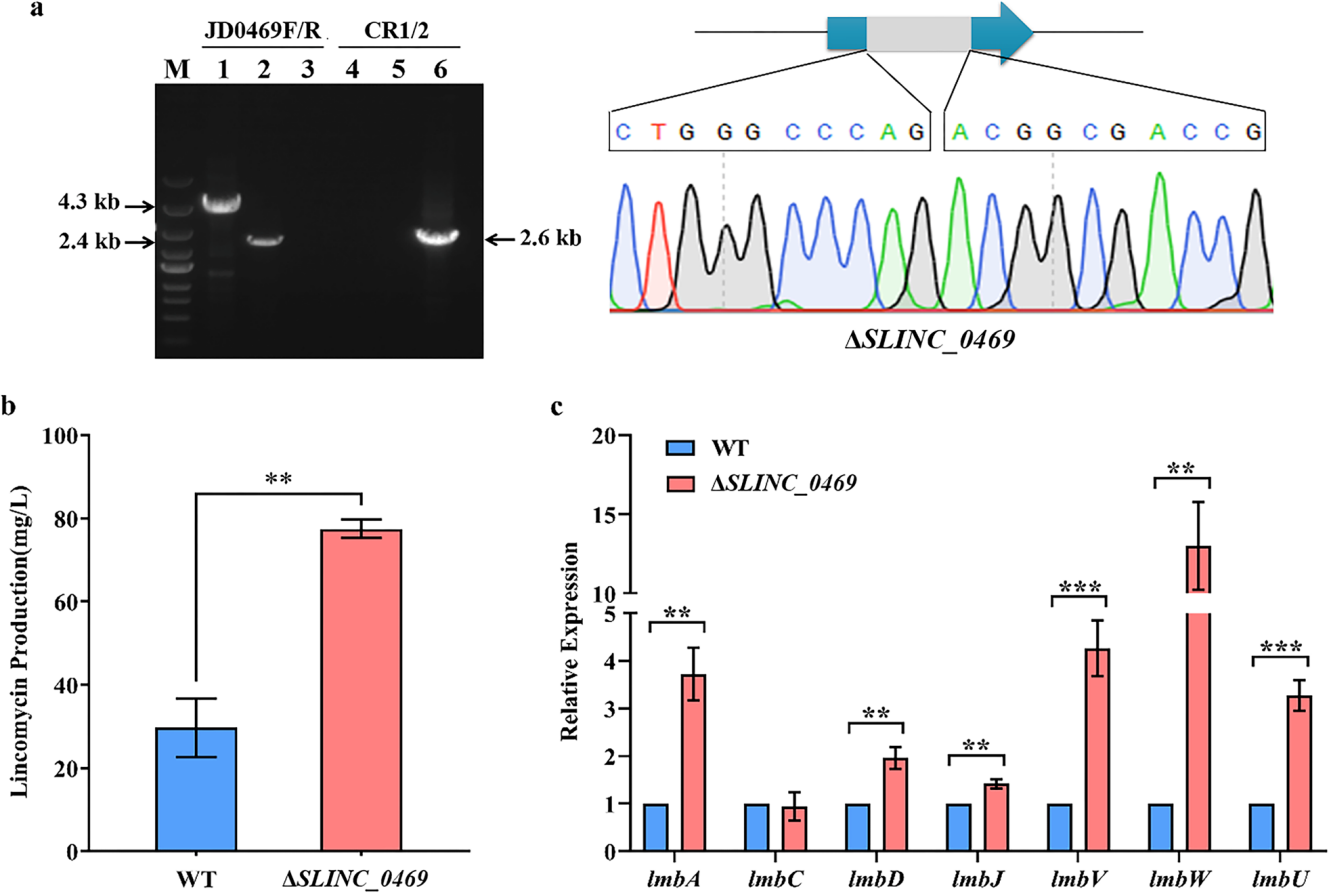
SLINC_0469 suppresses lincomycin biosynthesis. **a** Identification of Δ*SLINC_0469* by PCR and sequencing. Lane M indicated the DNA molecular weight marker. Lanes 1, 2 and 3 indicated PCR products amplified by primer pair JD0469F/R. Lanes 4, 5 and 6 indicated PCR products amplified by primer pair CR1/CR2. 1 and 4, WT; 2 and 5, Δ*SLINC_0469*; 3 and 6, pKCcas9d0469. **b** Effect of SLINC_0469 on lincomycin production. **c** Transcriptional analysis of lincomycin biosynthetic genes in WT and Δ*SLINC_0469*. The relative expression was normalized using internal reference gene *hrdB*. The transcriptional level of each gene in WT was set to 1.0. **P < 0.01; ***P < 0.001

To further investigate the function of SLINC_0469 in *S. lincolnensis*, a *SLINC_0469* disruption strain Δ*SLINC_0469* was constructed. The mutant Δ*SLINC_0469* was confirmed by PCR using the primer pair JD0469-F/R (Fig. 4a). PCR products of WT with intact *SLINC_0469* gene and Δ*SLINC_0469* with defective *SLINC_0469* gene were 4.3 kb (Fig. 4a Lane 1) and 2.4 kb (Fig. 4a Lane 2) respectively. PCR amplification by primer pair CR1/CR2 was used to determine that the disruption plasmid pKCcas9d0469 was eliminated from the mutant Δ*SLINC_0469*. A 2.6 kb band appeared only using pKCcas9d0469 as template (Lane 6), rather than WT (Lane 4) or Δ*SLINC_0469* (Lane 5). Moreover, sequencing analysis verified that the mutant Δ*SLINC_0469* was constructed successfully (Fig. 4a). Subsequently, a *SLINC_0469* complementation strain C*SLINC_0469* was also constructed.

The result showed that inactivation of *SLINC_0469* had no significant influences on cell growth (Additional file 1: Fig. S4). Then, the WT, Δ*SLINC_0469*, C*SLINC_0469* strains were cultured in FM2 medium to measure the lincomycin production. The results showed that the yield of lincomycin in Δ*SLINC_0469* increased 2.6-fold compared to that in WT (Fig. 4b), and complementation of *SLINC_0469* restored lincomycin production (Additional file 1: Fig. S7a), indicating that SLINC_0469 negatively regulates lincomycin biosynthesis.

Furthermore, qRT-PCR analysis was carried out to assess the influence of SLINC_0469 on transcription of *lmb* genes. There are 8 putative operons in the lincomycin cluster, and the first gene of each operon was chosen to perform qRT-PCR, except for *lmbK*, which could not be detected due to the low transcriptional level according to our previous study [27]. Compared to WT, the transcriptional levels of *lmbA*, *lmbV*, *lmbW* and *lmbU* were significantly increased in Δ*SLINC_0469* with fold changes 3.7, 4.3, 13.0 and 3.3, respectively (Fig. 4c). Similar transcriptional levels of *lmbC*, *lmbD* and *lmbV* were observed in WT and Δ*SLINC_0469*, suggesting *lmbC*, *lmbD* and *lmbV* were not regulated by SLINC_0469. These data demonstrated that SLINC_0469 can suppress the transcription of *lmbA*, *lmbV*, *lmbW* and *lmbU*, thereby inhibiting lincomycin biosynthesis.

### SLINC_1037 negatively regulates lincomycin biosynthesis

The 1908-bp *SLINC_1037* gene encodes an AcoR family transcriptional regulator. A conserved Walker A motif (**G**ERGT**GK**) and a HTH motif were found in the internal and the C-terminal of SLINC_1037 respectively (Additional file 1: Fig. S5a, b), indicating SLINC_1037 has putative ATP-binding and DNA-binding activities.

**Fig. 5:**
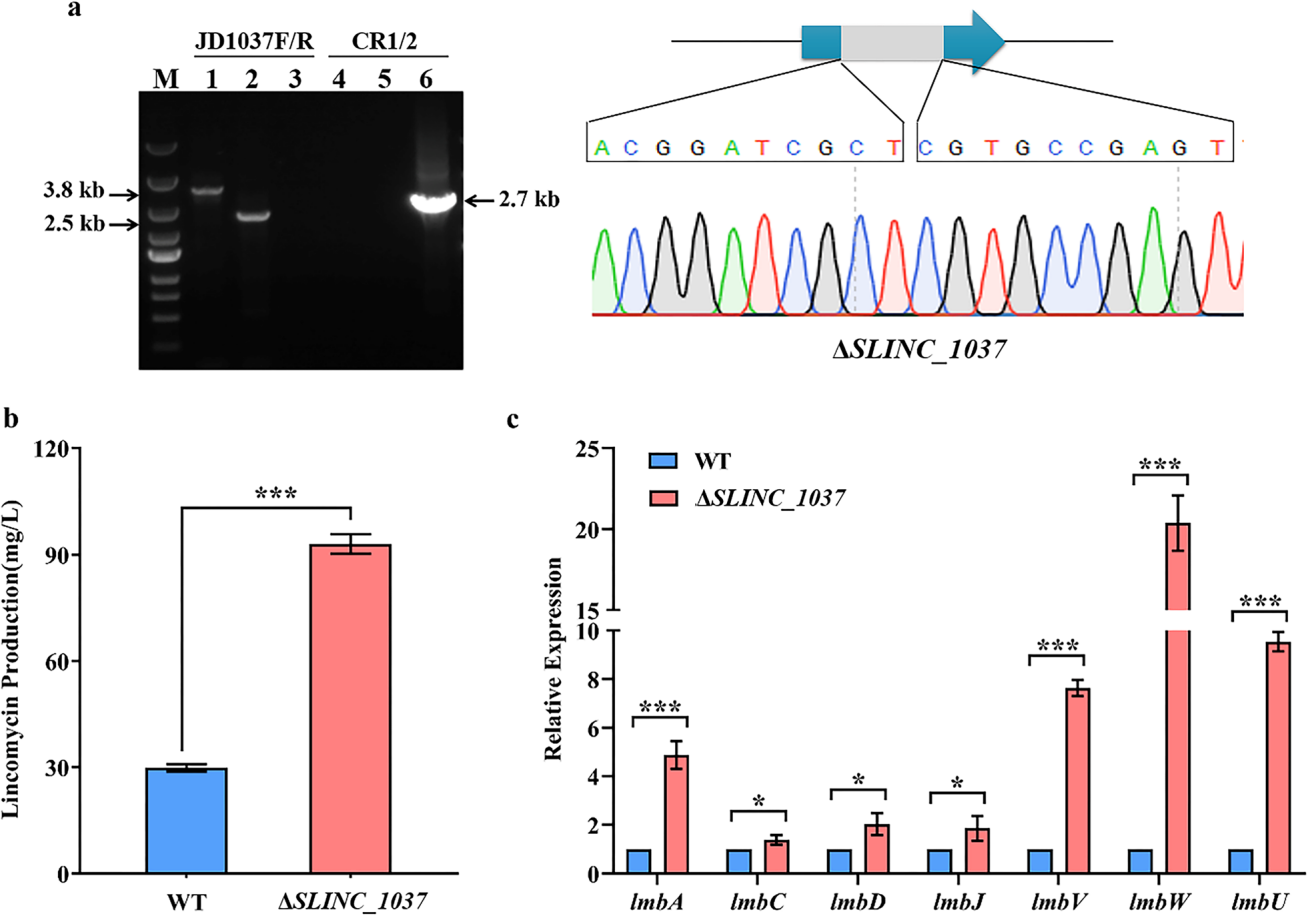
SLINC_1037 suppresses lincomycin biosynthesis. **a** Identification of Δ*SLINC_1037* by PCR and sequencing. Lane M indicated the DNA molecular weight marker. Lanes 1, 2 and 3 indicated PCR products amplified by primer pair JD1037F/R. Lanes 4, 5 and 6 indicated PCR products amplified by primer pair CR1/CR2. 1 and 4, WT; 2 and 5, Δ*SLINC_1037*; 3 and 6, pKCcas9d1037. **b** Effect of SLINC_1037 on lincomycin production. **c** Transcriptional analysis of lincomycin biosynthetic genes in WT and Δ*SLINC_1037*. The relative expression was normalized using internal reference gene *hrdB*. The transcriptional level of each gene in WT was set to 1.0. *P < 0.05; ***P < 0.001

Then, *SLINC_1037* disruption strain Δ*SLINC_1037*, complementation strain C*SLINC_1037* was constructed using the method as above. PCR products amplified by the primer pair JD1037-F/R were 3.8 kb and 2.5 kb with WT and Δ*SLINC_1037* as templates, respectively (Fig. 5a). Inactivation of *SLINC_1037* had no significant influences on cell growth (Additional file 1: Fig. S4). The results of lincomycin bioassays showed that the yield of lincomycin in Δ*SLINC_1037* increased 3.1-fold compared to that in WT (Fig. 5b), and complementation of *SLINC_1037* restored lincomycin production (Additional file 1: Fig. S7b), indicating that SLINC_1037 negatively regulates lincomycin biosynthesis. qRT-PCR analysis revealed that, compared to WT, the transcriptional levels of *lmbA*, *lmbV*, *lmbW* and *lmbU* were significantly increased in Δ*SLINC_1037* with fold changes 4.9, 7.6, 20.4 and 9.5, respectively (Fig. 5c). These data demonstrated that SLINC_1037 can suppress transcription of *lmbA*, *lmbV*, *lmbW* and *lmbU*, thereby inhibiting lincomycin biosynthesis.

### SLINC_8097 negatively regulates lincomycin biosynthesis

The 906-bp *SLINC_8097* gene encodes an AraC family transcriptional regulator, the C-terminal of which contains an AraC type DBD with putative DNA-binding activity (Additional file 1: Fig. S6a). Structure modeling and sequence alignment revealed that the DBD of SLINC_8097 includes similar HTH motifs referred to the template structure of AdpA (SMTL ID: 3w6v.1) [45], and the amino acids are conserved in *Streptomyces* (Additional file 1: Fig. S6B).

**Fig. 6:**
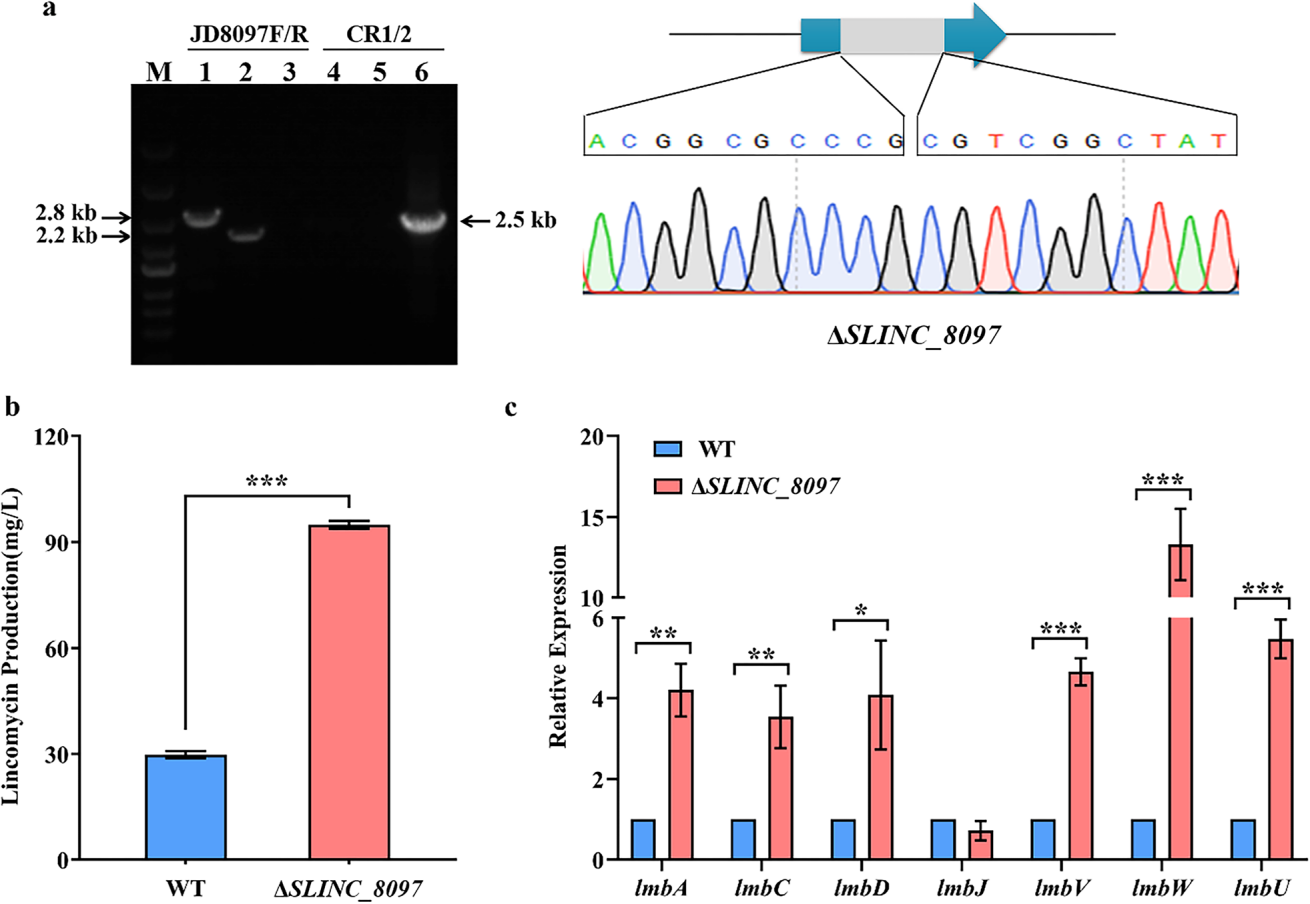
SLINC_8097 suppresses lincomycin biosynthesis. **a** Identification of Δ*SLINC_8097* by PCR and sequencing. Lane M indicated the DNA molecular weight marker. Lanes 1, 2 and 3 indicated PCR products amplified by primer pair JD8097F/R. Lanes 4, 5 and 6 indicated PCR products amplified by primer pair CR1/CR2. 1 and 4, WT; 2 and 5, Δ*SLINC_8097*; 3 and 6, pKCcas9d8097. **b** Effect of SLINC_8097 on lincomycin production. **c** Transcriptional analysis of lincomycin biosynthetic genes in WT and Δ*SLINC_8097*. The relative expression was normalized using internal reference gene *hrdB*. The transcriptional level of each gene in WT was set to 1.0. *P < 0.05; **P < 0.01; ***P < 0.001

Then, *SLINC_8097* disruption strain Δ*SLINC_8097*, complementation strain C*SLINC_8097* were constructed. PCR products amplified by the primer pair JD8097-F/R were 2.8 kb and 2.2 kb with WT and Δ*SLINC_8097* as templates, respectively (Fig. 6a). Inactivation of *SLINC_8097* had no significant influences on cell growth (Additional file 1: Fig. S4). The results of lincomycin bioassays showed that the yield of lincomycin in Δ*SLINC_8097* increased 3.2-fold compared to that in WT (Fig. 6b), and complementation of *SLINC_8097* restored lincomycin production (Additional file 1: Fig. S7c), indicating that SLINC_8097 negatively regulates lincomycin biosynthesis. Furthermore, qRT-PCR analysis demonstrated that, compared to WT, the transcriptional levels of *lmbA*, *lmbC*, *lmbD*, *lmbV*, *lmbW* and *lmbU* were significantly increased in Δ*SLINC_8097* with fold changes 4.2, 3.5, 4.1, 4.7, 13.3 and 5.5, respectively (Fig. 6c). The transcriptional levels of *lmbJ* were similar in WT and Δ*SLINC_8097*, suggesting *lmbJ* was not regulated by SLINC_8097. These data demonstrated that SLINC_8097 can suppress the transcription of *lmbA*, *lmbC*, *lmbD*, *lmbV*, *lmbW* and *lmbU*, thereby inhibiting lincomycin biosynthesis.

## Discussion

**Fig. 7:**
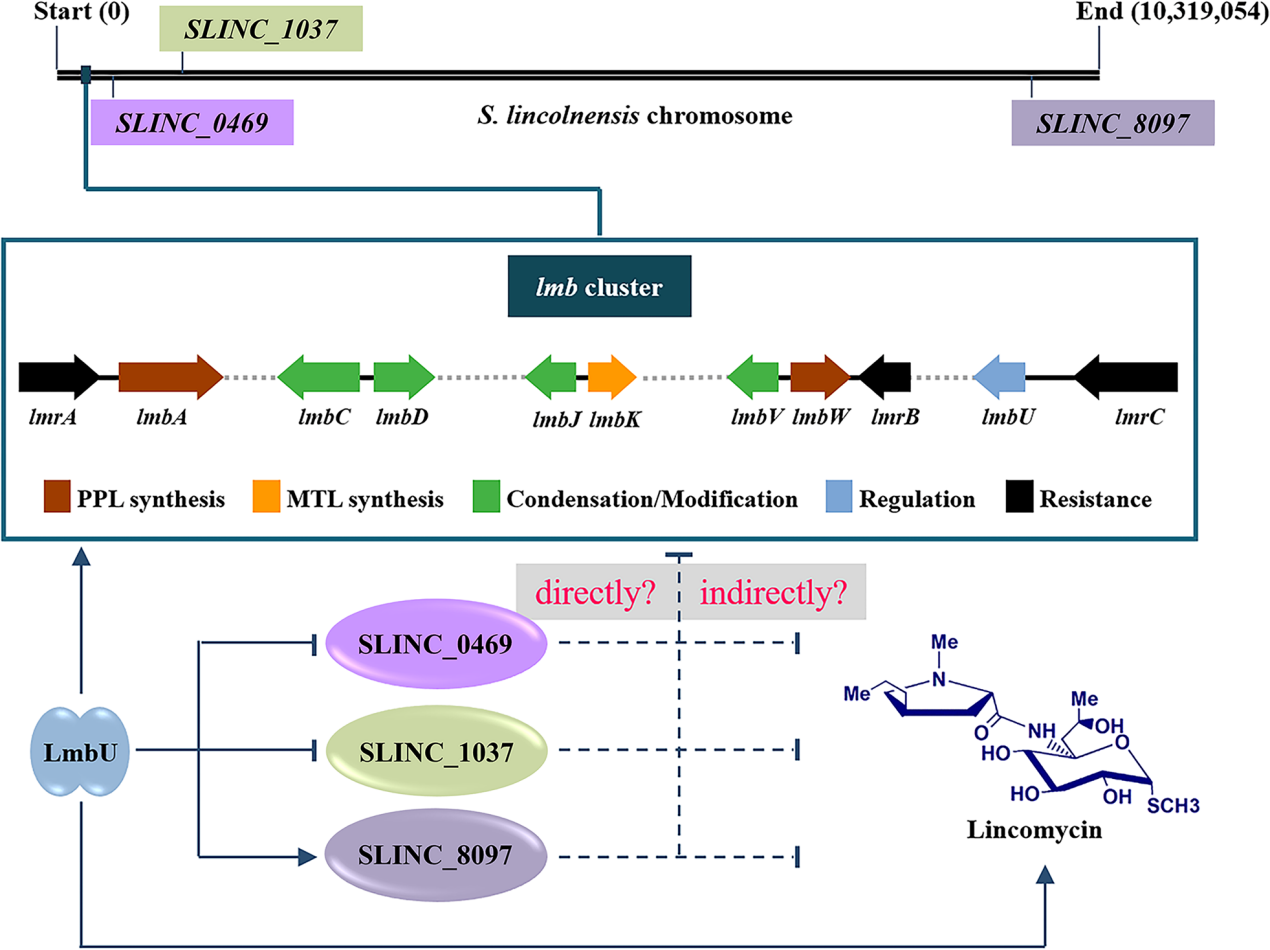
Proposed model of LmbU mediating regulation network to lincomycin biosynthesis. The locations of the *lmb* cluster and the three target genes on the chromosome were indicated. The arrows indicate activation, and the vertical virgules indicate inhibition. The solid lines indicate direct actions; the dotted lines indicate unknown mechanisms

Cross-regulation of CSRs among disparate antibiotic biosynthetic pathways has been widely studied in *Streptomyces* [13]. However, it is rarely reported that CSRs regulate the targets outside the BGCs. Here, we found that the DBSs of LmbU are widely distributed in the genome of *S. lincolnensis*, suggesting that LmbU is likely to function as a pleiotropic regulator and regulate more targets except the *lmb* genes (Fig. 7). Furthermore, through comparative transcriptomic analysis, Lin et al. [46] revealed that LmbU could regulate the transcription of 20 non-*lmb* genes, acting as a pleiotropic transcriptional regulator. It is worth noting that, the three targets of LmbU reported in our study, *SLINC_0469*, *SLINC_1037* and *SLINC_8097*, were not included in the regulated genes identified by Lin’s RNA-seq data [46]. The *S. lincolnensis* strain NRRL 2936 used in our study is wild type, while the strain SyBE2901 used in RNA-seq analysis is a high lincomycin producer. Furthermore, we conducted additional studies to provide evidence that the targets of LmbU can regulate the *lmb* genes and affect the production of lincomycin, which forms a cascade regulatory network.

Here, we used the conserved DBS of LmbU to screen the targets of LmbU, and performed EMSAs to investigate whether LmbU can bind to the targets directly. Though the sequence of DBS of LmbU within each target is perfectly matched, the binding affinity is not similar, indicating that the flanking sequence of DBS or the structure of the DNA may be also important for DNA-binding of LmbU. As reported, two ways have been found to be involved in DNA sequences recognition of proteins [47]. One way is directly based on contacts of amino acids and bases, and the flanking sequences are also important [48]. For example, the first and second flanking positions 5ʹ to the consensus DBS play important roles in DNA-binding affinity for E12 homodimer and E12-TAL1 heterodimer [49]. The other way is indirectly mediated by the conformation of the DNA [50–52]. Thus, the structures of DNA, including bendability, stability, groove shape, flexibility and so on, rather than the simple sequence are more appropriate to determine the DNA-binding affinities of proteins.

According to the study, LmbU inhibited the transcription of *SLINC_0469* and *SLINC_1037*, which negatively regulate the production of lincomycin, suggesting that LmbU affect the production of lincomycin not only by activating the *lmb* genes, but also by suppressing the genes against lincomycin biosynthesis. On the contrary, LmbU activates the transcription of *SLINC_8097*, which negatively regulates the production of lincomycin. This may be conducive to maintain the level of lincomycin within a certain range in vivo. These data revealed that the regulatory network of LmbU on lincomycin biosynthesis is complex and accurate. In addition, LmbU can bind to the promoter regions of *SLINC_0585* (encode an MFS transporter), *SLINC_6382* (encode an ABC transporter permease), *SLINC_7298* (encode an MFS transporter), *SLINC_6570* (encode an RNA polymerase sigma factor) and *SLINC_1077* (encode a methyltransferase). The studies about the regulatory mechanisms of LmbU to these targets and the effects of these target genes on lincomycin biosynthesis are ongoing.

## Conclusion

In our previous studies, we have demonstrated that LmbU functions as a CSR of lincomycin, and LmbU homologues are widely found in actinomycetes, but their positions on the chromosome are not limited to the antibiotic BGCs [27, 44]. Based on this, we screened and identified the targets of LmbU which are located outside the *lmb* cluster, and showed the effect of these targets on production of lincomycin. The data elucidated that LmbU promotes lincomycin biosynthesis not only through regulating transcription of the *lmb* genes, but also by regulating three target genes outside the *lmb* cluster. In addition, the three targets SLINC_0469, SLINC_1037 and SLINC_8097 have been found negatively regulated lincomycin biosynthesis via regulating transcription of the *lmb* genes including *lmbU*, forming cross-regulation. This study can further illuminate the regulatory network of lincomycin biosynthesis, and will bring light to the functional analysis of LmbU family regulators.

### Supplementary Information

**Additional file 1: Fig. S1. **EMSA of His_6_-LmbU (0, 3.2, 6.4 and 9.6 µM) with the negative probe. **Fig. S2. **Construction and identification of *S. lincolnensis* *lmbU* disruption mutant Δ*lmbU*. **Fig. S3. **Functional domains and sequence alignment of SLINC_0469. **Fig. S4. **Growth curves of *S. lincolnensis *strains NRRL 2936, Δ*SLINC_0469*, Δ*SLINC_1037*, and Δ*SLINC_8097*. **Fig. S5. **Functional domains and sequence alignment of SLINC_1037. **Fig. S6. **Functional domains and sequence alignment of SLINC_8097. **Fig. S7. **Effect of three new LmbU targets towards lincomycin production. **Table S1. **Primers used in this study.

## Data Availability

The datasets generated and analyzed during the current study are available from the corresponding author on reasonable request.

## Abbreviations

CSRG(s): Cluster-situated regulatory gene(s)
CSR: Cluster-situated regulator
BGC: Biosynthetic gene cluster
*lmb*: Lincomycin biosynthetic gene
LB: Luria–Bertani media
MS: Mannitol-soya flour
YEME: Yeast extract-malt extract
PCR: Polymerase chain reaction
bp: Base pair
Cas9: CRISPR-associated protein 9
CRISPR: Clustered Regularly Interspaced Short Palindromic Repeats
qRT-PCR: Quantitative real-time PCR
EMSA: Electrophoretic mobility shift assay
HTH: Helix-turn-helix
DBD: DNA-binding domain
DBS: DNA-binding site
