# Supplementary material for: Three new LmbU targets outside lmb cluster inhibit lincomycin biosynthesis in Streptomyces lincolnensis
Source: Microb Cell Fact. 2024 Jan 3;23:3. doi: 10.1186/s12934-023-02284-y (PMC10763038; doi:10.1186/s12934-023-02284-y)
Supplement: Supplementary file 1 — Additional file 1: Fig. S1. EMSA of His6-LmbU (0, 3.2, 6.4 and 9.6 µM) with the negative probe. Fig. S2. Construction and identification of S. lincolnensis lmbU disruption mutant ΔlmbU. Fig. S3. Functional domains and sequence alignment of SLINC_0469. Fig. S4. Growth curves of S. lincolnensis strains NRRL 2936, ΔSLINC_0469, ΔSLINC_1037, and ΔSLINC_8097. Fig. S5. Functional domains and sequence alignment of SLINC_1037. Fig. S6. Functional domains and sequence alignment of SLINC_8097. Fig. S7. Effect of three new LmbU targets towards lincomycin production. Table S1. Primers used in this study. [file 12934_2023_2284_MOESM1_ESM.docx]

**Supplementary materials**

**Three new LmbU targets outside *lmb* cluster inhibit lincomycin biosynthesis in *Streptomyces lincolnensis***

Yue Mao^1, 2^, Xianyan Zhang^1, 2^, Tianyu Zhou^1, 2^, Bingbing Hou^1, 2^, Jiang Ye^1, 2^, Haizhen Wu^1, 2 *^, Ruida Wang^1, 2 *^, Huizhan Zhang^1, 2^

1 State Key Laboratory of Bioreactor Engineering, East China University of Science and Technology, Shanghai, China

2 Department of Applied Biology, East China University of Science and Technology, Shanghai, China

* Corresponding authors: wuhzh@ecust.edu.cn (Haizhen Wu); biord726@163.com (Ruida Wang)

**Table of Contents:
Fig. S1.** EMSA of His_6_-LmbU (0, 3.2, 6.4 and 9.6 µM) with the negative probe.

**Fig. S2.** Construction and identification of *S. lincolnensis* *lmbU* disruption mutant Δ*lmbU*.

**Fig. S3.** Functional domains and sequence alignment of SLINC_0469.

**Fig. S4.** Growth curves of *S. lincolnensis* strains NRRL 2936, Δ*SLINC_0469*, Δ*SLINC_1037*, and Δ*SLINC_8097*.

**Fig. S5.** Functional domains and sequence alignment of SLINC_1037.

**Fig. S6.** Functional domains and sequence alignment of SLINC_8097.

**Fig. S7.** Effect of three new LmbU targets towards lincomycin production

**Table S1.** Primers used in this study.

**Fig. S1.** EMSA of His_6_-LmbU (0, 3.2, 6.4 and 9.6 µM) with the negative probe.

**
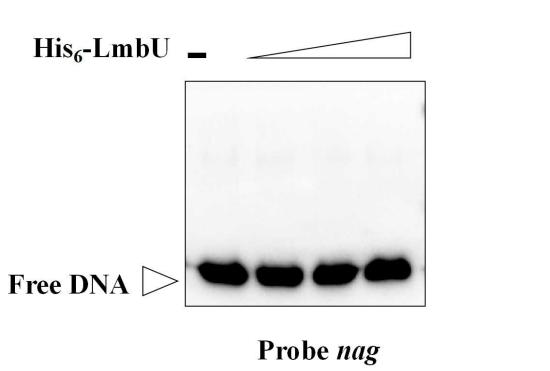
**

**Fig. S2.** Construction and identification of *S. lincolnensis* *lmbU* disruption mutant Δ*lmbU*. **a.** Schematic representation of *lmbU* disruption. The internal region of *lmbU* was deleted by CRISPR/Cas9-based genetic editing method. U and D indicated the *lmbU*-upstream and *lmbU*-downstream regions. The primer pairs CR1/CR2 and JDU-F/R were indicated by arrows. **b.** Identification of Δ*lmbU* by PCR. Lane M indicated the DNA molecular weight marker (DL2503, Generay, Shanghai, China). Lanes 1, 2, 3 and 4 indicated PCR products amplified by primer pair JDU-F/R. Lanes 5, 6, 7 and 8 indicated PCR products amplified by primer pair CR1/CR2. 1 and 5, WT; 2, 3, 6 and 7, Δ*lmbU*; 4 and 8, pKCcas9dlmbU. **c.** Sequencing analysis of the chromosome of Δ*lmbU*.

**
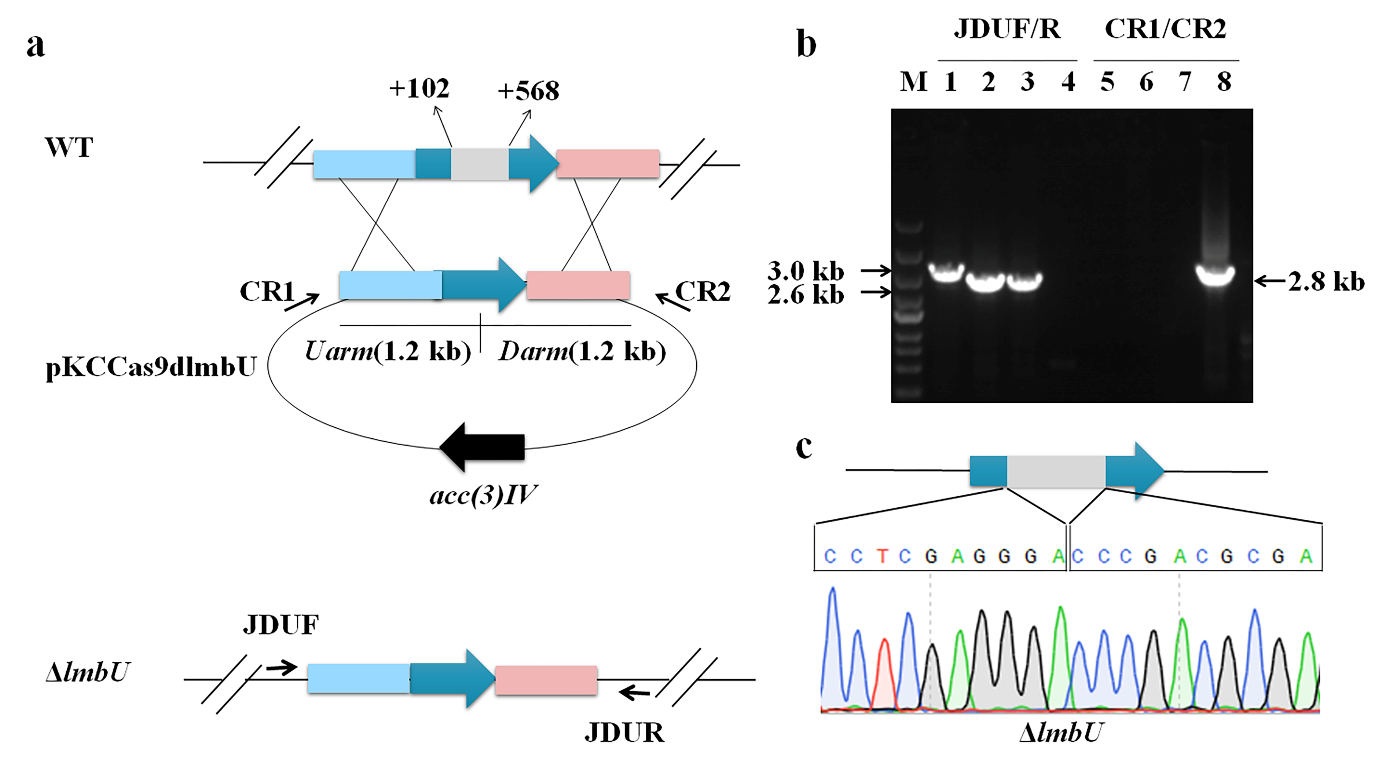
**

**Fig. S3.** Functional domains and sequence alignment of SLINC_0469. **a** Predicted domains of SLINC_0469. AAA^+^: ATPases Associated with a wide variety of Activities; HTH: helix-turn-helix motif of the LuxR family for DNA binding. **b** Alignment of the AAA domain of SLINC_0469 with related proteins. **c** Comparison of the HTH domain of SLINC_0469 with that of other proteins. 1, SLINC_0469 from *S.* *lincolnensis* (WP_067426176.1); 2, AveR from *Streptomyces* *avermitilis* (BAA84600.1); 3, FkbN from *Streptomyces tsukubensis* (TAI41675.1); 4, GdmRI from *Streptomyces* *hygroscopicus* (ABI93791.1) 5, GdmRII from *Streptomyces* *hygroscopicus* (ABI93788.1); 6, PikD from *Streptomyces venezuelae* (AAC68887.1); 7, RapH from *Streptomyces* *hygroscopicus* (AAC38065.1); 8, SalRI from *Streptomyces albus* (ABG02267.1); 9, TtmRI from *Streptomyces ahygroscopicus subsp. wuzhouensis* (AFW98290.1); 10, TtmRII from *Streptomyces ahygroscopicus subsp. wuzhouensis* (AFW98288.1). The conserved amino acids of Walker A and Walker B are indicated by red asterisk.

**
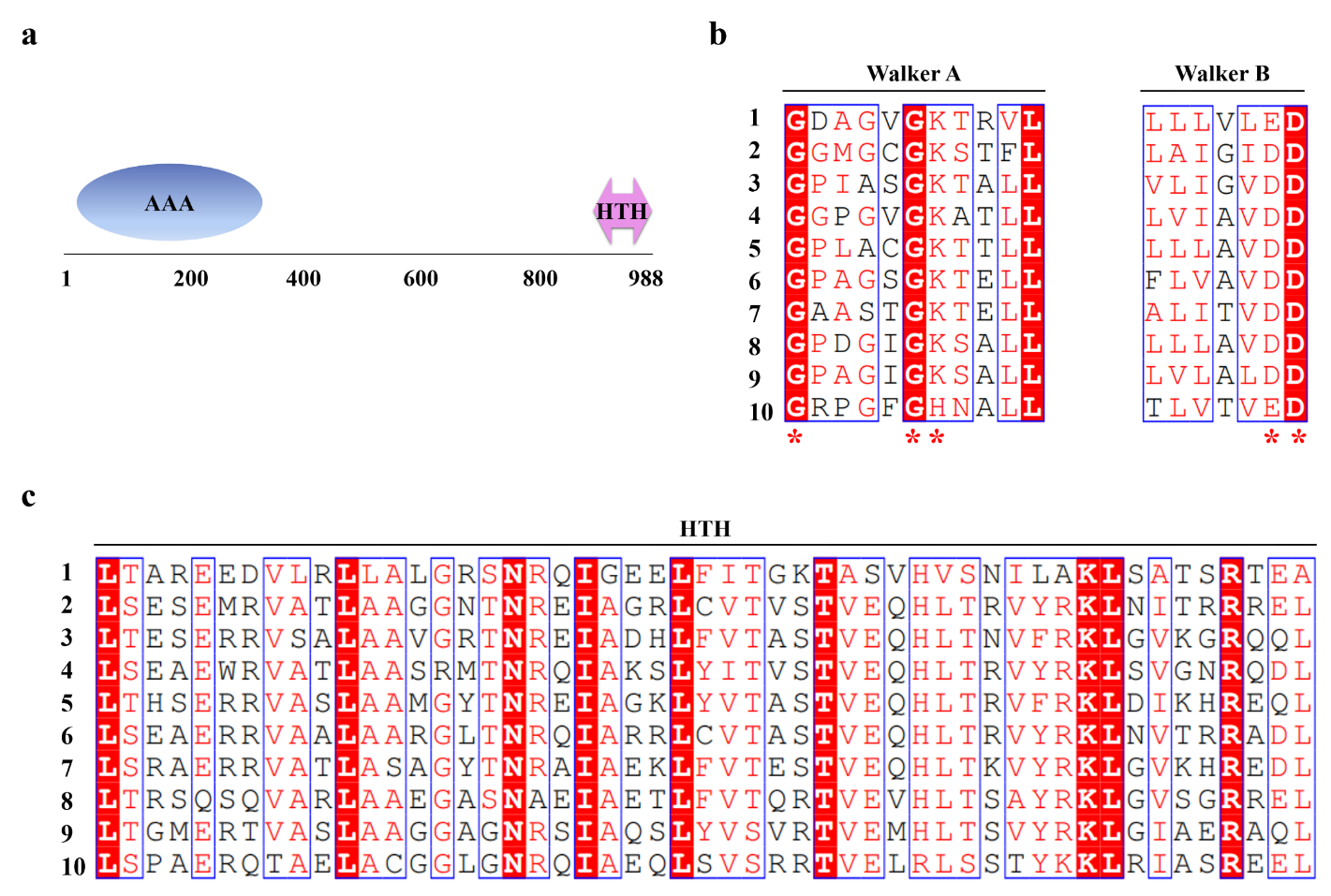
**

**Fig. S4.** Growth curves of *S. lincolnensis* strains NRRL 2936, Δ*SLINC_0469*, Δ*SLINC_1037*, and Δ*SLINC_8097*.

**
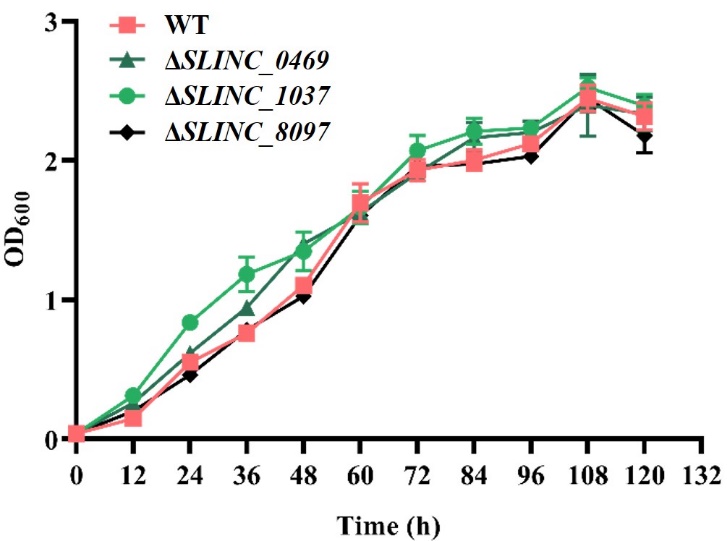
**

**Fig. S5.** Functional domains and sequence alignment of SLINC_1037. **a** Predicted domains of SLINC_1037. **b** Alignment of the HTH motif of SLINC_1037 with that of related proteins. 5m7n.1, the template used for structure modeling of SLINC_1037 in SWISS-MODEL. 1, SLINC_1037 from *S.* *lincolnensis* (WP_079164420.1); 2, *Streptomyces aurantiogriseu* (WP_189940635.1); 3, *Streptomyces fulvoviolaceus* (WP_078655870.1); 4, *Streptomyces dysideae* (WP_079085070.1); 5, *Streptomyces scabiei* (WP_037704052.1); 6, *Streptomyces bluensis* (GGZ64430). The conserved amino acids of Walker A are indicated by red asterisk.

**
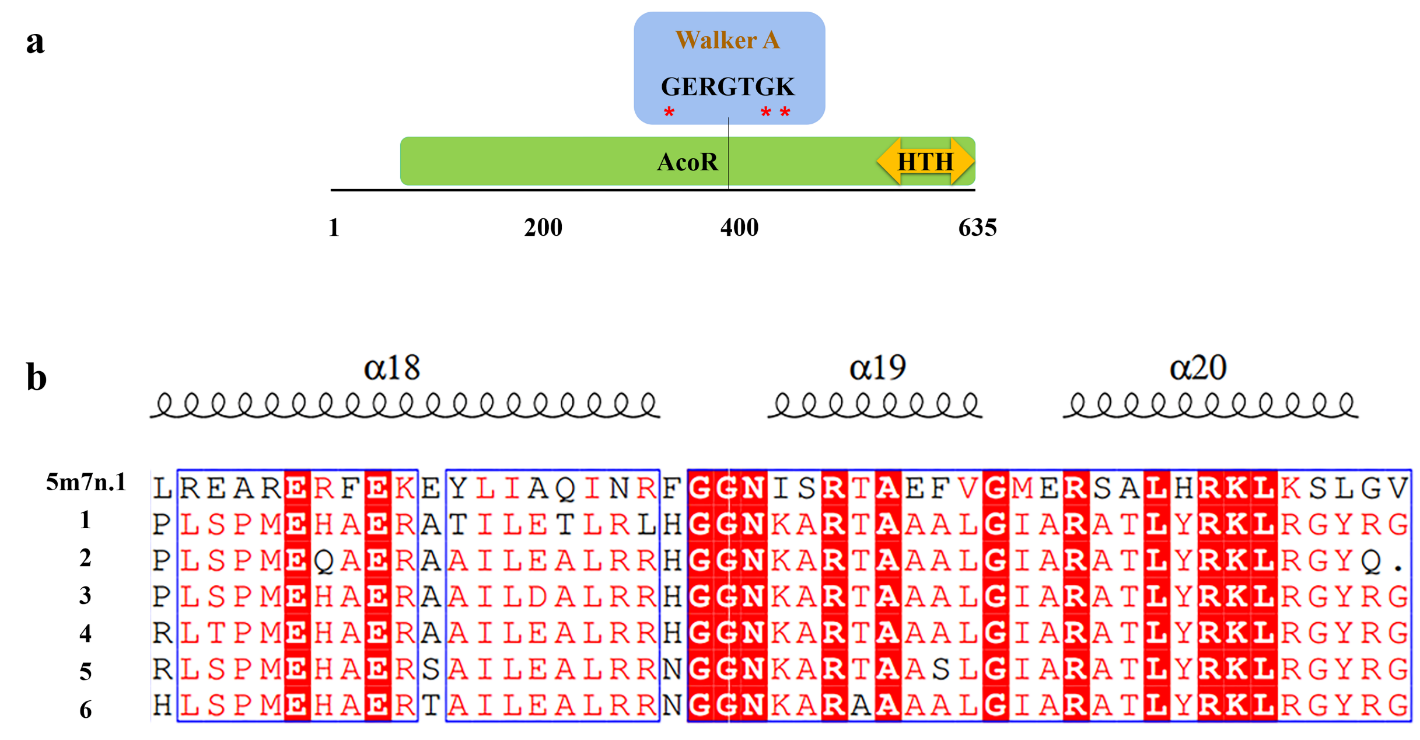
**

**Fig. S6.** Functional domains and sequence alignment of SLINC_8097. **a** Predicted domains of SLINC_8097. **b** Alignment of the DBD domains of SLINC_8097 with related proteins. 3w6v.1, the template used for structure modeling of SLINC_8097 in SWISS-MODEL. 1, SLINC_8097 from *S. lincolnensis* (WP_067443797.1); 2, *Streptomyces albicerus* (WP_151477398.1); 3, *Streptomyces albiflavescens* (WP_189192684.1); 4, *Streptomyces canu*s (WP_059211104.1); 5, *Streptomyces davaonensis* (WP_015663102.1); 6, *Streptomyces scabichelini* (WP_165261112.1).

**
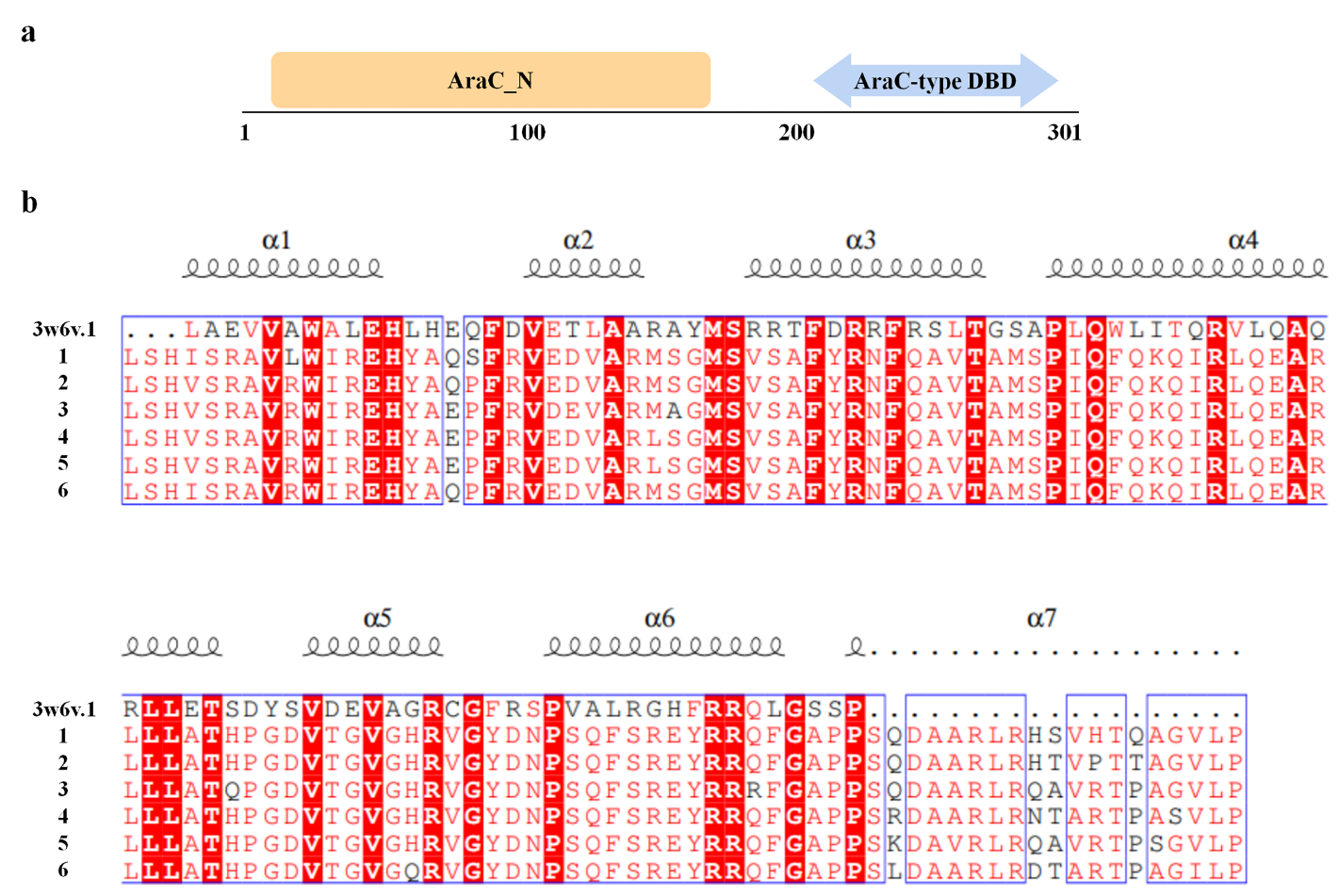
**

**Fig. S7.** Effect of three new LmbU targets towards lincomycin production **a** Lincomycin production of ΔSLINC_0469, CSLINC_0469. **b** Lincomycin production of ΔSLINC_1037, CSLINC_1037. **c** Lincomycin production of ΔSLINC_8097, CSLINC_8097. The results were achieved from two independent experiments. *, *P* < 0. 1; **, *P* < 0.01; ***, *P* < 0.001.

**
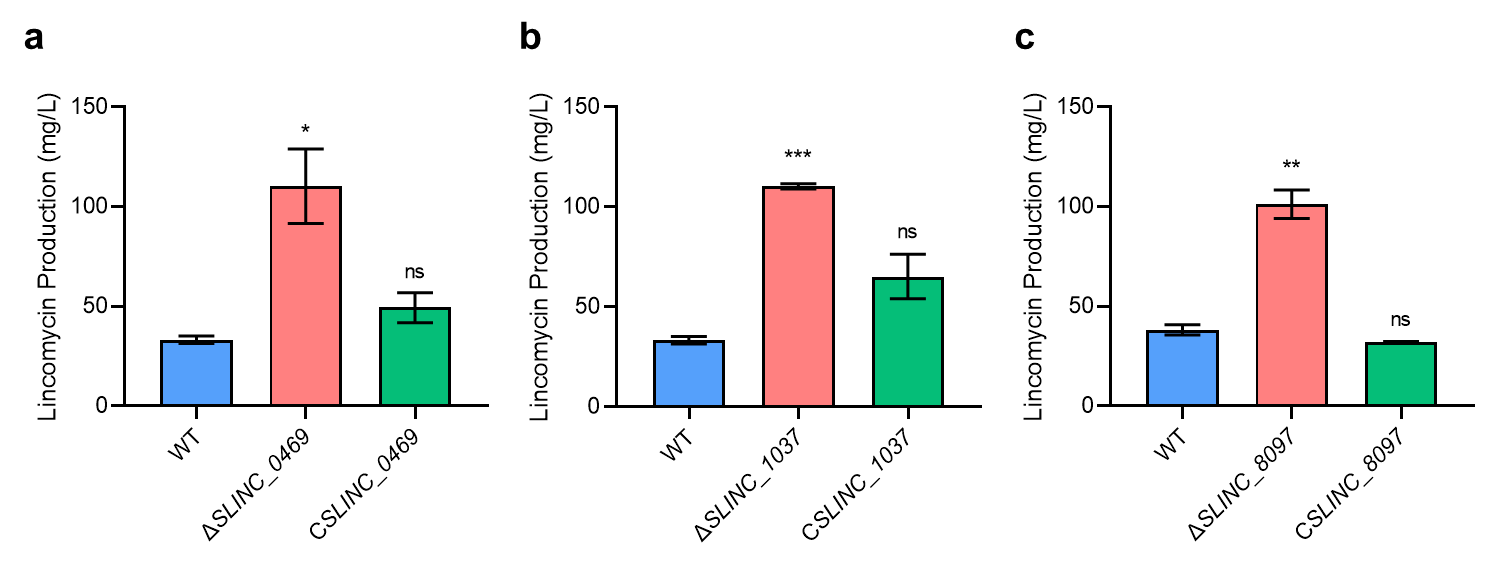
**

**Table S1.** Primers used in this study.

| Primers | Sequence (5’ to 3’) |
| --- | --- |
| EMSA | |
| UBS-1037-F | AGCCAGTGACGATAAGGGTGCTGGGGCATGTCG |
| UBS-1037-R | AGCCAGTGACGATAAGGAGGTGCTGCGGGACCTC |
| UBS-37445-F | AGCCAGTGACGATAAGGGCGTGCCATAGGGGTTC |
| UBS-37445-R | AGCCAGTGACGATAAGCCGACATGGCCGACGACTAC |
| UBS-0585-F | AGCCAGTGACGATAAGAGGTGGTCAAGGCGGTCT |
| UBS-0585-R | AGCCAGTGACGATAAGGTGTCCTGCTGAGCGTGG |
| UBS-1746-F | AGCCAGTGACGATAAGTCTTGGTGTCCGCCGTCG |
| UBS-1746-R | AGCCAGTGACGATAAGGGCTACCAGCGCTTCGA |
| UBS-6382-F | AGCCAGTGACGATAAGCGTACCCGGCGTACTCGA |
| UBS-6382-R | AGCCAGTGACGATAAGGCTGAGCACGCTGAAGGAG |
| UBS-4366-F | AGCCAGTGACGATAAGGCGGCGTACCTGACGGATTA |
| UBS-4366-R | AGCCAGTGACGATAAGGGCGACCCGGAGAACAA |
| UBS-6271-F | AGCCAGTGACGATAAGACCCTGCCGCTGCTCG |
| UBS-6271-R | AGCCAGTGACGATAAGCGGCCTTGTAGGTGCG |
| UBS-6570-F | AGCCAGTGACGATAAGGAACTGCTGCGGCTGGT |
| UBS-6570-R | AGCCAGTGACGATAAGCGACGTGGACGTTGCG |
| UBS-1077-F | AGCCAGTGACGATAAGCGATGATGTCGGTGCTCCC |
| UBS-1077-R | AGCCAGTGACGATAAGGCGGCCAGGATCTCCAAG |
| UBS-6232-F | AGCCAGTGACGATAAGTCGTGTCGGCCAGCACATC |
| UBS-6232-R | AGCCAGTGACGATAAGGCGGCACTGACCATCAGC |
| UBS-4499-F | AGCCAGTGACGATAAGGCCGGATTCCTCCAGGCG |
| UBS-4499-R | AGCCAGTGACGATAAGCCAGTCCGCACCCAAGCC |
| UBS-0469-F | AGCCAGTGACGATAAGGGCGGCGGATGGTGGTTC |
| UBS-0469-R | AGCCAGTGACGATAAGCGTCGCGAAGTTCCAGTACC |
| UBS-7298-F | AGCCAGTGACGATAAGGCGGTGACCTCGTCGTTG |
| UBS-7298-R | AGCCAGTGACGATAAGTTCGGCGTGGGGATGGA |
| UBS-8097-F | AGCCAGTGACGATAAGCGAACACTGCGTCGTCTACC |
| UBS-8097-R | AGCCAGTGACGATAAGGCACCAGGGGATCTGCC |
| *nag*-F | AGCCAGTGACGATAAGGGCCACACGTCGTAGACATCTGCG |
| *nag*-R | AGCCAGTGACGATAAGCGCCGTCCCGGTCGAAGAAC |
| EMSA-B* | Biotin-AGCCAGTGACGATAAG |
| Construction of disruption mutant Δ*lmbU* | |
| sgUF | CGGACTAGT*CGTTGCGCCTCAGCTCGTTG*GTTTTAGAGCTAGAAATAGCAAGTTAAAAT |
| sgUR | CGGTAGCGGACTTCGGAATTCGCAGATCTCAAAAAAAGC |
| uU-F | **GAGATCTGCGAATTC**CGAAGTCCGCTACCGCCTG |
| uU-R | **ACTCCTCGAGGGA**CCCGACGCGAGTGACAAGTACA |
| dU-F | **CACTCGCGTCGGG**TCCCTCGAGGAGTGGATTTGCG |
| dU-R | CCCAAGCTTATCTCGAACGGGACCACGAT |
| JDUF | CGGCTCGGGCAAGTCAA |
| JDUR | TCGGGCACCACCGTCAA |
| CR1 | GGTGCAAGCCCGGACGTT |
| CR2 | CCATTCAGGCTGCGCAACT |
| *xylTE* reporter assays | |
| p0469-F | ***CTCTTCGCTATTACGCCAG***TCGTCGTCACCAAGCACCA |
| p0469-R | **CCTCGTAGCCGGCACTGTTCAT**GGATCCATCCTGGCACGTGAGAC |
| p1037-F | ***CTCTTCGCTATTACGCCAG***TGGACGAAGGACTTGTAGACG |
| p1037-R | **CCTCGTAGCCGGCACTGTTCAT**TCCCTGGAGCGTTACCGTG |
| p8097-F | ***CTCTTCGCTATTACGCCAG***CGGTGTACTCGGACTCCTTGGT |
| p8097-R | **CCTCGTAGCCGGCACTGTTCAT**GTCCCCATTGCAGCACCATG |
| pAxyl-3 | ATGAACAGTGCCGGCTACGAGG |
| pAxyl-4 | ***GGCCGATTCATTAATGCAG***TCAGGTCAGCACGGTCATGAATC |
| Construction of disruption mutants Δ*0469*, Δ*1037* and Δ*8097* | |
| sg-0469 | ***CAGTCCTAGGTATAATACTAG****TGGGTCGGCAGTTGCTCGTC*GTTTTAGAGCTAGAAATAGCAAGTTAAAATAAGGCTAGTCCGTTATC |
| u-0469-F | **TAGCAAGTTAAAATAAGGCTAGTCCGTTATCAACTTGAAAAAGTGGCACCGAGTCGGTGC**CGACCCGTTCTTCACCACC |
| u-0469-R | **GCAGTTCGGTCGCCGTCTG**GGCCCAGTGCAGATCCTCC |
| d-0469-F | **CTGCACTGGGCC**CAGACGGCGACCGAACT |
| d-0469-R | ***ACGACGGCCAGTGCCAAGCT***GTGATCAGGATCGCTGTCATC |
| sg-1037 | ***CAGTCCTAGGTATAATACTAG****ACTCGTCAGTCTCGTCAGCC*GTTTTAGAGCTAGAAATAGCAAGTTAAAATAAGGCTAGTCCGTTATC |
| u-1037-F | **TAGCAAGTTAAAATAAGGCTAGTCCGTTATCAACTTGAAAAAGTGGCACCGAGTCGGTGC**CGATGAGCCACTCGTTCCAGG |
| u-1037-R | **TCGGGCAACTCGGCACG**AGCGATCCGTTCGAGCACC |
| d-1037-F | **GAACGGATCGCT**CGTGCCGAGTTGCCCGACC |
| d-1037-R | ***ACGACGGCCAGTGCCAAGCT***GCTGGTGGTCGTGGCTGT |
| sg-8097 | ***CAGTCCTAGGTATAATACTAG****CCTGGAGCCGGATCTGCTTC*GTTTTAGAGCTAGAAATAGCAAGTTAAAATAAGGCTAGTCCGTTATC |
| u-8097-F | **TAGCAAGTTAAAATAAGGCTAGTCCGTTATCAACTTGAAAAAGTGGCACCGAGTCGGTGC**CTCCTCGTTCCGTCAGTCC |
| u-8097-R | **TGTACGAGTACGGCGCC**CGCGTCGGCTATGACAACCC |
| d-8097-F | **TAGCCGACGCG**GGCGCCGTACTCGTACACCC |
| d-8097-R | ***ACGACGGCCAGTGCCAAGCT***TCGCCCATCACCATCCC |
| JD0469F | GTCCACGCCATGAACAGCAC |
| JD0469R | CTTCCTCAGCGGAGACAAACC |
| JD1037F | CGCCGAGGAGGACTTGATG |
| JD1037R | ATGATTTCCGATACGAGATGCC |
| JD8097F | ACCAGGGCAACACCAGCATG |
| JD8097R | GCCGGTAGTCCACCGAGACG |
| Construction of complementation strain C*SLINC_0469*, C*SLINC_1037*, C*SLINC_8097* | |
| 0469-R | GGCCGATTCATTAATGCAGcgggccctggcggctca |
| 1037-R | GGCCGATTCATTAATGCAGtcagccccggtagccgc |
| 8097-R | GGCCGATTCATTAATGCAGtcagggaaggacgcctgcct |
| qRT-PCR | |
| *hrdB*-RTF | GGGCCTTCGAAGCTGACC |
| *hrdB*-RTR | TGGCCGGACTCTTCGCT |
| *lmbA*-RTF | CGACACCGCAAGCCTTCTCCGAT |
| *lmbA*-RTR | CGAGCAACCGCAGCCAGCCAC |
| *lmbC*-RTF | CGGATAGGGCACGGAGAGCCATAC |
| *lmbC*-RTR | CCCCGCTGCACTTCGACGTGTC |
| *lmbJ*-RTF | AGCGACGGGATCGTGTTTG |
| *lmbJ*-RTR | CCTGGTCCTTCAGTGCCTCA |
| *lmbV*-RTF | CCCACCAGCACCGTCATG |
| *lmbW*-RTF | CGGTTCCCGCACCAGAAGA |
| *lmbW*-RTR | GCTGCGTGAGGACGTGGATG |
| *lmbU*-RTF | GGTGTCGGACTTCTTCTGCCC |
| *lmbU*-RTR | CGCAACTACGCCTGGGTGG |
| *lmbD*-RT-F | GGTCCTGAGCCTGCGTCT |
| *lmbD*-RT-R | CGCGCACAGGTACTCCCA |

*Italics*, N20 of sgRNA; **bold**, sequences homologous to corresponding homologous arm or sgRNA; ***bold and Italics***, sequences homologous to pKCcas9dO or pSET152; underlining, restriction enzyme cutting site.
